# Some Modern Continental Hospitals

**Published:** 1908-11-28

**Authors:** 


### HOSPITAL ADMINISTRATION.

### CONSTRUCTION AND ECONOMICS.

### SOME MODERN CONTINENTAL HOSPITALS.

#### THE MOABITE HOSPITAL, BERLIN.—II.

There is not much surgical work done at this hospital, but one of the pavilions is reserved for out-patient work, and formerly pavilion No. 12 was used as an operating theatre. The surgical side was found to be in need of enlargement, and it was therefore decided to build a proper theatre. The City Council in 1895 voted a special grant for its construction, and the building was finished in July 1896. It is a double-storied block, situated near the pavilions used for surgical patients, but not connected with these by means of a corridor. On the ground floor is the main operating-room, 9 by 7.25 metres, airy and exceedingly well lighted; a second room for septic cases, 6.75 by 4.25 metres; and a third and smaller room, 6 by 4 metres, for out-patient work. All three rooms have sky and side lights. Side light is insured by large windows, which can be turned at right angles, rotating as they do upon vertical axes, and thus permitting excellent ingress of light and air. For night work several open burners and reflecting arc lamps, the former no longer in use, are provided. The floors, and the walls up to a height of three metres, are tiled, and the usual precautions to insure efficient cleaning by "flushing" corners and rounding edges have been observed. The instrument cases are of glass and enamel ware; the tables of simple construction, but efficient and strong. These theatres cannot claim to be regarded as ideal up-to-date types, but they have the merit of being simple and are quite efficient for the work that is done. Next to the theatre for out-patient work is a large sterilising room with boilers for normal saline and distilled water, and the usual disinfecting apparatus. On the ground floor are, in addition, a plaster room, a bandage room, a bath room, very complete lavatory accommodation, a waiting room, an anæsthetising room, a laboratory for microscopical and centrifugalising work, and an *x*-ray room. A feature is the excellently lighted room for throat and nose work. On the first floor are the attendants' rooms and bandage rooms.

In 1900 the large new kitchens were built, and the "economic block" is well worth inspection. Compared with the gigantic establishment at the Virchow it is small, but compared with any of our London institutions it is a magnificently arranged and excellently constructed department. In outside appearance this block conforms to the style that has been adopted for the rest of the departmental buildings, and is very similar to the laundry block which lies opposite it. The main kitchen is a large tiled apartment, 16 by 12 metres, containing fourteen large nickel-plated soup boilers, ovens, and extra apparatus. Smaller rooms are arranged on the same ground floor. Thus there is a very large vegetable room, where it is a sight to watch the electric potato peeling machines, the cabbage slicers, and the choppers at work. Close to it is the sausage room, an institution in every German hospital which may be commended to the consideration of our hospital authorities. The economic advantages of sausages as an article of hospital diet are real, and as manufactured at the Moabite the sausages are excellent variants in the daily menu of patients on full diet, supplying to a large extent the place of the unappetising "mince" which is so prominent a feature in London hospitals. Another institution is the room where that favourite German dish *Nudeln* is turned out in large quantities daily. Further on one comes to the refrigerator and meat chambers. Here hang rows upon rows of sheep and bullock carcasses, and there is a special pork and game room. The institution buys only carefully selected meat, which must conform to a very high standard, be delivered in whole carcasses, of half sides not exceeding a special weight limit, and be freshly killed. These carcasses are "hung" for a certain time, as is the case in large hotels or at Simpson's. The result is that the meat supplied is exceptionally good, as, indeed, is all the food. The London hospital patient will be surprised to find the excellent menu which his German friend in distress obtains when on full diet. Expressed on paper it may seem ordinary, but the cooks ring the changes in so skilful a fashion, the meals are served in so appetising a manner, and the viands are so good, that the visitor who does not go into figures, or who is ignorant of the strict economy practised, will probably regard the hospital as a most extravagant institution. The diet scale at the Moabite may not be without interest to Hospital readers :—

Diet Scale.

| Class | Morning and Afternoon | Midday | Evening | Whole Day |
|---|---|---|---|---|
| 1 | ½ litre milk<br>½ litre milk-coffee | $\frac{9}{10}$ litre vegetables, with boiled or fried fish, fowl or game, red meat, steak, or potatoes<br>½ litre soup | Sausage, red or white meat, fish, potatoes, eggs, cheese<br>½ litre soup | 250 grms. bread, 50 gr. butter or fresh dripping, 150 gr. milk roll, two bottles beer (for male adults only) |
| 2 | ½ litre milk or milk-coffee | ¾ litre vegetables, with extras as above, excluding pork, game, or fowl | Milk soup, with milk rolls, *Nudeln* | 250 gr. bread, 100 gr. milk rolls, 50 gr. butter |
| 3 | ½ litre milk or milk-coffee | Broth | Vermicelli, &c. | 50 gr. biscuits |
| 4 | ½ litre milk | Milk, chocolate, sago | Milk soup | — |
| 5 | Special diet as ordered by physician in special cases. | | | |

The patients' food is put in specially designed, solid metal food-carriers, which are warmed in hot-water wagons and carried to the various pavilions.

There is a large housekeeper's room where special food is stored, rolls and salami cut, and the spices are kept. Next to it is the egg room, in which the large stock of eggs is preserved in large wooden cabinets with perforated shelves. This arrangement enables the housekeeper to see almost at a glance when the stock needs replenishing. Alongside is a special cool chamber for milk. On the first floor are the dwelling rooms for the *personnel*. The kitchen maids have their rooms here, being lodged in large, airy and well-furnished rooms, each with two or four beds. Like every other part of the hospital, these rooms were scrupulously clean, and their large windows, overlooking the park, admitted plenty of light and air. No washhand basins are to be found in these rooms, there being a large common lavatory on the same floor. There the visitor may note a specially designed gas apparatus for heating curling tongs. "If we don't give them this," said the superintendent with a smile, "they will use the gas burners." The water-closets are excellently arranged and similar to those in the wards. The married attendants have special apartments on this and the next floor.

In the laundry, a fine double-storied block lying on the right-hand side, are to be seen many special contrivances, besides the full electric washing plant. Most of the apparatus are models of the types supplied by the well-known Duisburg firm of Martin Bros. In the sorting-room is a series of strong wire cages labelled "Handkerchiefs," "Sheets," "Shirts," etc., into which the dirty linen is sorted as soon as it enters the department. This considerably facilitates the work of checking the washing, as a special shift is responsible for each lot. The large metal trolleys used for conveying the lots to and from the various rooms are simple and strong, and a special "Moabite model." Double connections are present with each piece of machinery, in case there is a failure of current, and the work can be carried on by alternating sets of dynamos. In the clean-linen rooms there are again sorting chests of drawers and shelves. Here, too, is a large seamstresses'-room, where some twenty seamstresses and sewing maids are employed. All the material used is of the best, as it has been found to be more economical in the long run. The machine-room has specially constructed boilers which supply the heating power for the various buildings. In winter five to seven of these are working day and night; in the summer months two suffice. The heating is by high-pressure steam in the kitchens, operating-room, laundry, and disinfecting department; elsewhere low-pressure steam is used. The pipes are isolated, and have copper condensing tubing. The loss of heat in the main system has been found to be less than $\frac{1}{4}$ atmosphere for 1,000 metres distance.

The institution does not possess a full electro-therapeutic or hydrotherapeutic department at present, but it is proposed to build one as soon as possible. The water supply is largely from the city water pipes, but there is a special reservoir which makes the hospital independent of the main system should any shortage occur. The disinfecting department is fully up to date, and possesses one of the finest steam-disinfecting plants of any Continental hospital. Dry steam is used, and it has been found to be thoroughly efficient at a temperature of 125° C. maintained for an hour.

The administrative block, which is comparatively new, having been completed in 1906, contains the usual offices and the dwelling-rooms of the staff. To the extreme right of it is the nurses' home and nursing school. This is a three-storied building, which was erected in 1895, and is thoroughly suited for its purpose. It was modified and enlarged in 1906, several new bathrooms being added and the sanitary arrangements thoroughly overhauled. The building accommodates 130 nurses, probationers, and ward-maids. The day-rooms are large and cheerful, the main dining-room, with its outlook upon the park, being especially roomy. The sisters or head nurses possess each a large bed-sitting room, well furnished, and on the ground floor. The probationers are housed in smaller separate rooms on the first floor, and the wardmaids have four- and six-bedded dormitories on the top floor. Probationers must be below 35 years of age and not less than 20 years old, of good moral character and general education, and must pass a strict medical examination before they are admitted. There are no fees, and the probationer receives board and lodging free, but she must agree to serve for three years in one of the city hospitals on completion of her course. After six months' strict probation she is promoted to a ward as junior nurse, receiving 10s. a month pocket-money. At the end of her course, lasting from a year to eighteen months, she has to pass an examination, and is then promoted to ward nurse (or sister, though the *Oberschwester* or head sister really corresponds to the sister in an English ward) at a salary of 360 marks per annum, rising by an annual increment of 50 marks to 540 marks. When she has served three years as nurse she is promoted to head nurse or sister at a maximum salary of 660 marks yearly. Provision is made for pension fund, as by regulation of the City Council a sum not exceeding one quarter of the total yearly income for each year of service is granted the retiring nurse after a period of not less than ten years' service. For pension purposes the yearly cost of board and lodging is added to the nominal salary that the probationer receives, being reckoned as equivalent to about £50, and in each case the calculation is made from the day the probationer enters the school.

At the Moabite they have effectually prevented gross abuse *by refusing to take any patient free of charge.* Each patient pays according to a fixed scale; adults, if belonging to the city, pay 2.50 marks per day; if residing outside the city limit 3 marks; children are charged 2.50 marks daily, or if from parts beyond Berlin 3 marks. Poor patients, of whom the hospital annually treats a large number, are no exception to this rule. Their fees are paid for them by the city authorities on the following conditions: (1) That the patient is virtually too poor to pay the small fee charged, a point which is investigated by the police and municipal authorities, who institute very stringent inquiries; (2) that the patient has been certified as being in need of hospital treatment by one of the recognised "Physicians for the Poor" in Berlin, and (3) that the patient has not been attending elsewhere or been discharged from another hospital for good reasons. All these conditions are very important ones, and I need only take the third to point out how welcome some such scheme of rejection of "black-list" patients would be in London. A fourth condition exists on paper, namely, that the poor patient must belong to one of the city wards in the immediate neighbourhood of the Moabite. The result of this in-patient system is that in many of the wards one may see citizens of good standing who are receiving such treatment as they would not be able to obtain in an English nursing home at more than ten times the price. It is no alleged disgrace to go to the Moabite, which in many respects and for several reasons (one of which is the fact that no venereal cases are admitted) is one of the most popular of Berlin hospitals. The patients are all "paying patients," but subject to ordinary hospital discipline, which is as strict as in any of our institutions. No autopsy is made, however, where patients' friends object. The hospital income is derived from seven different sources, which are tabulated as follows: Rent of vacant hospital ground; Donations, bequests, and interest on same; Special fees; Burial fees; Water Rate to private dwellings of staff; Sale of refuse; Patients' payments (in this the city grants for poor patients are not included); and finally the annual grants from the City Council paid out of the rates. During the last financial year the main figures for the Moabite were as follows: Sale of refuse, bones, straw, etc., 8,923 marks; patients' payments, 5,874 marks; total income from all sources, excluding the annual grants, 15,005 marks. In comparison with this one may give similar totals for the three other city hospitals, which are as follows: Friedrichshain, 12,763 marks; Urban, 13,450 marks; and Virchow, 5,535 marks. The total expenditure compares as follows: Moabite, 1,197,907 marks; Friedrichshain, 1,181,591 marks; Urban, 967,997 marks; Virchow (incomplete), 649,871 marks; or, if reduced to rate per patient per day, the figures in the same order are: 3.63 marks, 3.80 marks, 3.83 marks and 6.03 marks—a table in which the Moabite compares very favourably indeed with the other city hospitals, and exceptionally so with the Virchow hospital. Taking the expenditure more in detail, one finds that administrative expenses accounted for approximately 102,0C0 marks; wages for 93,000 marks.

The cost per capita of board works out very satisfactorily notwithstanding the exceptionally good menu that is given. Thus, for the very full table for the staff the cost is 2.10 marks per day; for table 2, at which nurses and administrative employees sit, the cost is 1.68; for the patients it is just below one mark, averaging for all three classes just over one mark. This low figure is due to the great economy and care that prevail in the kitchens, without which it would be impossible to board the inmates so excellently at so small a cost During the last financial year the hospital received 12,158 in-patients, as against the 13,000 at Friedrichshain and the 9,000 at Urban. Of this number 1,767 died and 9,562 were discharged, the mortality rate comparing very favourably with that of the other Berlin hospitals. The average daily number of occupied beds was 852 in 1906, and 795 in 1905; the average duration of patients' stay in hospital, twenty-three days; the average monthly return of new patients admitted, 861. The daily average of new patients admitted was 31, never falling below 29. These figures compare very well with those of any London hospital.

The direction of the hospital is vested in a superintendent director (*Verwaltungs-direktor*), who has charge of the administrative department, and two professional directors, one in charge of the surgical and the other at the head of the medical division. This is the arrangement that exists in all the communal hospitals, and which has been found to work exceedingly well. At the monthly meetings of the directors the physician in charge of the medical department is, by courtesy, the presiding head; in their respective departments the directors are independent and supreme, though the administrative director has necessarily a great deal to do with the wards. Expenditure up to an amount of approximately £8 may be sanctioned by the directors; above that sum the special authority of the hospitals committee of the City Council is necessary. Estimates are carefully checked, and, although there is no stinting of necessaries, waste and extravagance are sternly kept down.

Simple in its general plan, efficient in its work, and economically one of the best hospitals in the world, the Moabite Hospital is in every way a model of what the modern hospital should be. Criticisms, of course, one can bring against it in plenty. From a purely professional point of view the old pavilions, for instance, are too small and too simple in construction to be model types; they are, however, being rebuilt on the lines of the new types, and in a few years' time these objections will no longer hold. Generally speaking, the wards are excellent. I have already laid stress on their cheerfulness, perfect ventilation and good heating. As examples of what a good pavilion should be, the new wards are, indeed, excellent, and it is difficult to suggest in what way they may be modified without destroying the simplicity which is so characteristic a feature of the whole institution. The tendency towards architectural display which has been so strikingly shown in the Virchow hospital buildings is here seen in the new administrative block, but is much more excusable, as the hospital fronts the imposing new structures lying opposite it on the Turmstrasse side, and not, as in the case of the Virchow, a series of inartistic breweries. This floridity of building has necessarily added to the total cost of the hospital, which can no longer be regarded as the marvel of cheap hospital construction which it originally was; yet the total cost per bed, figuring out at somewhat below £100, is remarkably small. When the expenses for the rebuilding of the entire institution are added this figure will doubtless be very largely increased. As a general hospital the Moabite is well able to hold its own when compared with the better-known general institutions of the pavilion type. With its English rivals it compares favourably. Compared with the French and Italian hospitals the Moabite is immeasurably superior, both in its internal arrangements, its excellent nursing department, and its economical aspect. Its chief feature is throughout simplicity.

In conclusion we wish to express our thanks to Verwaltungs-direktor Borchardt for the great assistance his personal explanation of the various points of interest has been, and for his unfailing courtesy and kindness.

